# Glutamine Metabolism in Cancer Stem Cells: A Complex Liaison in the Tumor Microenvironment

**DOI:** 10.3390/ijms24032337

**Published:** 2023-01-25

**Authors:** Francesco Pacifico, Antonio Leonardi, Elvira Crescenzi

**Affiliations:** 1 Istituto per l’Endocrinologia e l’Oncologia Sperimentale, CNR, Via S. Pansini 5, 80131 Naples, Italy; 2 Dipartimento di Medicina Molecolare e Biotecnologie Mediche, University of Naples “Federico II”, Via S. Pansini 5, 80131 Naples, Italy

**Keywords:** glutamine, metabolism, cancer stem cells, tumor microenvironment, cancer-associated fibroblasts, adipocytes, senescent cells

## Abstract

In this review we focus on the role of glutamine in control of cancer stem cell (CSC) fate. We first provide an overview of glutamine metabolism, and then summarize relevant studies investigating how glutamine metabolism modulates the CSC compartment, concentrating on solid tumors. We schematically describe how glutamine in CSC contributes to several metabolic pathways, such as redox metabolic pathways, ATP production, non-essential aminoacids and nucleotides biosynthesis, and ammonia production. Furthermore, we show that glutamine metabolism is a key regulator of epigenetic modifications in CSC. Finally, we briefly discuss how cancer-associated fibroblasts, adipocytes, and senescent cells in the tumor microenvironment may indirectly influence CSC fate by modulating glutamine availability. We aim to highlight the complexity of glutamine’s role in CSC, which supports our knowledge about metabolic heterogeneity within the CSC population.

## 1. Glutamine Metabolism 

Glutamine (Gln) is a “conditionally” essential amino acid in cancer cells. In fact, although glutamine is a highly abundant amino acid with a plasma concentration of 0.5–0.6 mM in humans, glutamine demand in highly proliferative cancer cells can exceed both endogenous production and exogenous supply, inducing regional depletion within the tumor mass. In particular, areas of poor vascularization in the tumor show a selective deficiency of glutamine as compared to other amino acids, which are taken up less avidly by cancer cells [1]. Notably, tumor avidity for glutamine allows for imaging based on 18F-labeled glutamine tracers in preclinical and clinical studies [2]. 

**Figure 1:**
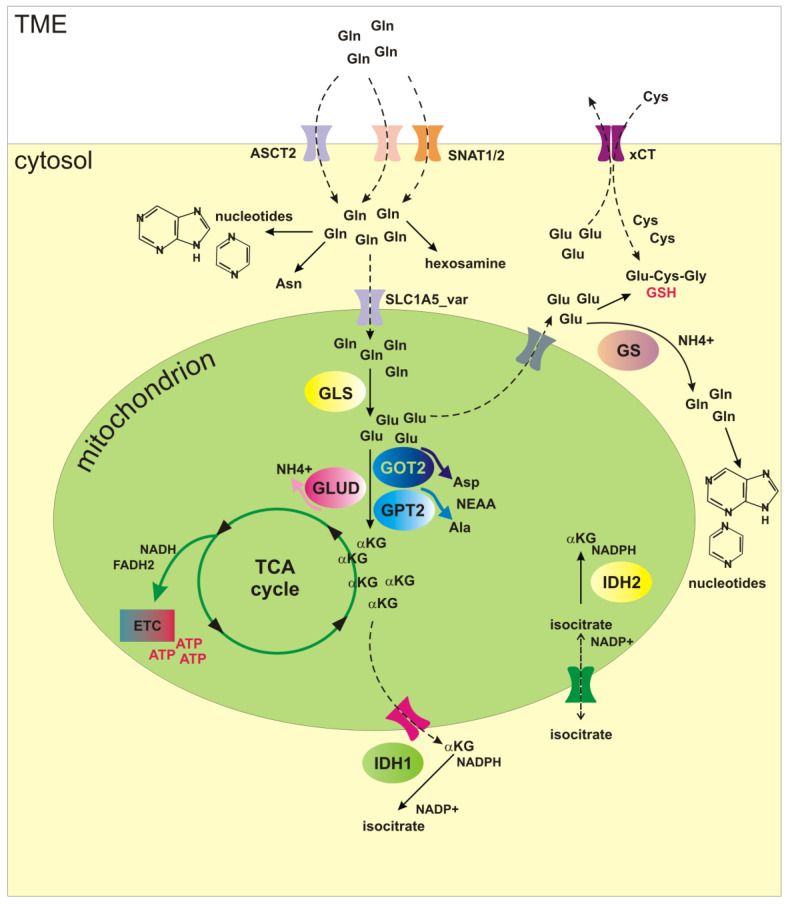
Overview of glutamine metabolism. Glutamine (Gln) is imported into the cytosol through various transporters (ASCT2, SNAT1, SNAT2). In the cytosol Gln acts as precursor for biosynthesis of asparagine (Asn), hexosamine and nucleotides. SLC1A5_var transports Gln into the mitochondria, where Gln is converted to glutamate (Glu) by glutaminase (GLS). Glu in the mitochondrial matrix is converted to α-ketoglutarate (αKG), either by glutamate dehydrogenase 1 (GLUD1) or by mitochondrial transaminases (GPT2, GOT2). Glu deamidation by GLUD1 is associated to production of ammonia (NH4+), whereas the conversion of Glu to αKG via transaminases is coupled to synthesis of non-essential amino acids (NEAA), aspartate (Asp) and alanine (Ala). GLS and transaminases are present both into the cytosol and into the mitochondria. The αKG enters the tricarboxylic acid (TCA) cycle, fuels the electron transport chain (ETC), and provides energy (ATP). Various mitochondrial carriers export Glu and αKG back to the cytosol. Cytosolic Glu is exported in exchange for cystine (Cys) by antiporter xCT. Cys and Glu are substrates for the synthesis of glutathione (GSH). Cytosolic isocitrate dehydrogenase IDH1 converts α-KG into isocitrate by oxidizing NADPH to NADP, whereas mitochondrial IDH2 converts isocitrate to αKG, and produces NADPH. Both IDH1 and IDH2 can catalyze the conversion between isocitrate and αKG, and the reverse reaction. Glutamine synthetase (GS) synthetize glutamine de novo through condensation of glutamate and NH4+ in the cytosol. αKG α-ketoglutarate; ETC electron transport chain; GLS glutaminase; GLUD1 glutamate dehydrogenase 1; GOT2 glutamic-oxaloacetic transaminase 2; GPT2 glutamic-pyruvic transaminase 2; GS glutamine synthetase; GSH reduced glutathione; IDH isocitrate dehydrogenase; NEAA non-essential amino acids; TCA tricarboxylic acid cycle; TME tumor microenvironment; xCT cystine-glutamate antiporter.

Glutamine uptake in cancer cells occurs through different transporters belonging to four different solute carrier (SLC) families [3]. Among them, ASCT2 (SLC1A5), SNAT1 (SLC38A1) and SNAT2 (SLC38A2) play a major role in cancer cells, since ASCT2 is required for optimal growth at low glutamine concentrations, whereas SNAT1 and SNAT2 mainly mediate net glutamine uptake for glutaminolysis [4]. Hence, ASCT2, SNAT1 and SNAT2 transport glutamine through plasma membrane into the cytoplasm, where glutamine acts as precursor substrate for synthesis of asparagine, hexosamine and purine/pyrimidine nucleotides (Figure 1). 

Further metabolism requires glutamine deamination by glutaminase (GLS) enzyme and can occur in the cytosol or in the mitochondria, thus modulating intracellular nitrogen distribution [5]. Glutamine transport from cytoplasm into the mitochondria is mediated by a recently identified variant of SLC1A5 (SLC1A5_var), transcribed from an alternative transcription initiation site, which plays a critical role in cancer metabolic reprogramming [6]. GLS initiates glutaminolysis by hydrolyzing glutamine into glutamate and ammonium. Mammalian cells express two GLS isoforms, namely kidney-type glutaminase (KGA or GLS1) and liver-type glutaminase (LGA or GLS2) [7] that are dysregulated in a cancer type-specific manner [8]. Furthermore, a GLS1 splice variant referred to as GAC or glutaminase C has been identified. Glutaminase C is exclusively localized to the mitochondria where it might be especially relevant for anaplerosis, whereas KGA is a cytosolic isoform [5]. 

Glutamate in the mitochondrial matrix is converted to α-ketoglutarate (αKG, 2-oxoglutarate), either by glutamate dehydrogenase 1 (GLUD1 or GDH1) or by different mitochondrial transaminases (GPT2, GOT2) [9]. Hence, GLUD and the mitochondrial transaminases compete for glutamate. Interestingly, it has been shown that these two pathways are differently used in proliferating vs. quiescent cells: proliferating cells preferentially convert glutamate to αKG via transamination reactions, while quiescent cells favor GLUD-dependent production of αKG. It is important to note that the conversion of glutamate to αKG via GPT2 or GOT2 is coupled to non-essential amino acids (NEAA) synthesis, specifically aspartate and alanine [10], which can be particularly important for growing cells. On the whole, these reactions deliver glutamine carbon to the tricarboxylic acid cycle (TCA), and so glutaminolysis represents a key anaplerotic reaction in cancer cells. Accordingly, glutamine can critically contribute to ATP production in transformed mammalian cells in which the TCA cycle is supplemented by glutamine-derived products instead of pyruvate from glycolysis [11]. Notably, glutaminolysis would support production of ATP not only via oxidative phosphorylation, but also through mitochondrial substrate level phosphorylation (mSLP), catalyzed by succinate-CoA ligase (SUCL), also known as succinyl coenzyme A synthetase. Indeed, SUCL can catalyze the conversion of succinyl-CoA and ADP to Coenzyme A, succinate and ATP, thereby compensating for ATP syntheses deficiencies frequently observed in cancer cells [12,13].

**Figure 2:**
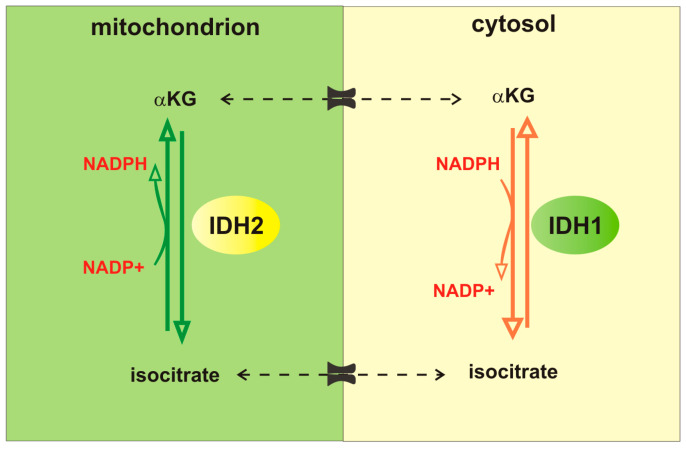
Isocitrate-αKG cycle modulates the NADP/NADPH balance. IDH1 is a cytosolic enzyme, IDH2 is mitochondrial enzyme. Both IDH1 and IDH2 catalyze the conversion between isocitrate and αKG, and the reverse reaction, i.e., the reductive carboxylation of αKG yielding to isocitrate. The forward reaction reduces NADP to NADPH, while the reverse reaction converts NADPH to NADP.

A series of transmembrane carriers allows for transport of interconvertible metabolic intermediates from cytosol to mitochondria and vice versa [14]. For instance, mitochondrial carriers can import glutamine, glutamate, and αKG to support the TCA cycle, but mitochondrial carriers can also export glutamate and αKG back to the cytosol [15]. Cytosolic glutamate is a substrate for the synthesis of glutathione (GSH), a key antioxidant molecule in cells [16]. Cytosolic glutamate is also a substrate for the cystine-glutamate antiporter xCT (SLC7A11), which imports extracellular cystine in exchange for glutamate, and plays an important role in antioxidant defense [17]. Finally, cytosolic, as well as mitochondrial glutamate, can be utilized as a precursor for the synthesis of NEAA aspartate, alanine, proline, and arginine [18]. Therefore, while on one side glutaminolysis increases intrinsic reactive oxygen species (ROS) production by fueling TCA cycle and the electron transport chain (ETC) [19], on the other hand glutamine-derived glutamate counteracts oxidative damage by inducing both cystine uptake and GSH synthesis. Furthermore, TCA intermediates, through a series of cyclical mitochondrial and cytosolic interconversions, generate NADPH, which is a donor of reductive potential used to maintain reduced glutathione pool and to neutralize ROS [14,20]. For instance, a citrate-isocitrate-αKG cycle modulates the NADP/NADPH balance. This cycle is activated by isocitrate dehydrogenases enzymes (IDH), which catalyze oxidative decarboxylation of isocitrate to αKG. Three different IDH proteins are found in cells, which differ for their localization and for enzymatic activity, and an extensive review has been recently published on these enzymes and their putative role in driving tumor progression [21]. Here, we will only summarize the differences between IDH1 and IDH2, which have been shown to regulate CSC metabolism and fate. In particular, IDH1 is a cytosolic enzyme, while IDH2 is expressed in mitochondria. Both IDH1 and IDH2 catalyze the conversion between isocitrate and αKG, and the reverse reaction, i.e., the reductive carboxylation of αKG yielding to isocitrate. Importantly, IDH1 and IDH2 use NADPH as a cofactor, so the forward reaction reduces NADP to NADPH, while the reverse reaction converts NADPH to NADP, linking IDH activity to cellular redox homeostasis (Figure 2).

Finally, cancer cells can synthetize glutamine de novo in an ATP-dependent reaction catalyzed by glutamine synthetase (GS; glutamate-ammonia ligase, encoded by GLUL gene), which drives condensation of glutamate and ammonia in the cytosol [22]. Activation of this important cataplerotic pathway is frequently observed in cancers and plays a critical role in promoting cell proliferation and survival under glutamine limitation [23,24,25]. Glutamine biosynthesis by GS also represents an important mechanism for elimination of ammonia in various tissues [26,27]. It is important to note that glucose, contributing to TCA cycle and αKG production, can be an important precursor in de novo glutamate synthesis, thereby supporting GS-dependent glutamine synthesis. In some cancers, during glutamine starvation, glucose increases its contribution to TCA cycle and plays a critical role replenishing glutamate pool [24]. 

## 2. Glutamine Metabolism in CSC

CSC represent a minor population of cancer cells endowed with both the ability to self-renew, thereby being able to regenerate the tumor, and to differentiate into a variety of different cell types, thus explaining the phenotypic and functional heterogeneity among tumor cells. In addition, CSC display high long-term tumorigenic potential upon injection in immunodeficient mice, have the ability to grow in vitro as spheres, and are resistant to conventional radiation and chemotherapy. It has also been suggested that CSCs could be responsible for metastasis [28,29]. Since CSC are able to regenerate tumors, they have also been termed tumor-initiating cells (TIC). According to their plastic behavior, different subsets of CSC can be identified within tumor mass that differ in proliferative ability, differentiation potential and expression of stem-specific markers [30,31]. CSC contribute to tumor progression, therapeutic resistance, and recurrence [32]. Notably, CSC have distinctive metabolic features and specific metabolic requirements, as compared with their differentiated progenies [33,34]. 

In recent years, glutamine and its downstream products have been shown to modulate CSC fate in different tissues, in a direct or indirect manner, and we will review these advances below, schematically separating studies according to the main metabolic pathways employed by CSC. We will show that CSC exhibit a complex response to glutamine variations, highlighted by opposing results reported in various studies.

### 2.1. Glutamine and Redox Homeostasis in CSC

The ability of glutamine to modulate redox homoeostasis through GSH synthesis and NADPH production plays a critical role in different CSC populations. Accordingly, interfering with glutamine metabolism has been shown to inhibit self-renewal and to decrease expression of stemness genes and pluripotency factors by increasing intracellular ROS [35,36,37,38,39]. However, molecular pathways and mechanisms through which glutamine metabolism modulates stemness are different in various cancer stem-like models. In human and murine embryonal carcinoma stem-like cells (ECSLC) a signaling network between TAp73, Myc, and SLC1A5 controls glutamine uptake and GSH biosynthesis [35]. So TAp73 controls levels of Myc-dependent SLC1A5 expression and maintains stemness by increasing cellular antioxidant defense. Accordingly, knockdown (KD) of TAp73 in ECSLCs induces ROS accumulation and reduces the expression of pluripotency factor OCT4, as well as the sphere-forming capacity of ECSLC. In line with a prominent role for redox control, both glutamine replenishment and the addition of antioxidants like N-acetyl-cysteine (NAC) or Mito-TEMPO restore cell growth in TAp73 KD ECSLC. Interestingly, in normal human embryonic stem cells glutamine metabolism has been shown to be essential to prevent oxidation-dependent degradation of OCT4 via GSH antioxidant activity [40]. A central role for GSH-mediated redox regulation has also been demonstrated in a stem-like cell subpopulation in triple-negative breast cancer (TNBC). This cell subset is characterized by increased expression of a recently described breast cancer stem cell marker, ganglioside GD2 [41], and depends on ASCT2-mediated glutamine uptake for glutathione synthesis and ROS homeostasis [36]. Interestingly, these cells are induced, both in vitro and in vivo in mice xenograft models, under metabolic stress (i.e., serum/glucose deprivation) via the conversion/dedifferentiation of GD2− into GD2+ breast cancer stem cells. In this CSC subset both pharmacologic inhibition with V9302 and knockdown of SLC1A5 result in simultaneous downregulation of GD2, reduced GSH content and increased ROS accumulation, associated with a significant inhibition of sphere-forming ability and tumorigenic capacity [36]. 

GLS-driven GSH biosynthesis also contributes to prostate cancer CSC maintenance and radioresistance [37]. Radioresistant isogenic sublines derived from prostate cancer cell lines DU145, LNCaP and PC3 display not only resistance to radiotherapy, but also increased stemness, defined by high aldehyde dehydrogenase (ALDH) activity, increased sphere-forming capacity in vitro and tumor-initiating potential in vivo. Targeted metabolomic analyses in these sublines reveal high intracellular levels of glutamate, which is converted to α-KG used in the synthesis of GSH. Accordingly, glutamine starvation or treatment with the GLS inhibitor CB-839 in these radioresistant cells decreases the GSH/GSSG ratio, increases ROS levels, activates an endoplasmic reticulum stress response, and induces apoptosis. A protective role of GLS-driven catabolism of glutamine is also confirmed in primary prostate cancer cells, which are sensitized to irradiation upon GLS inhibition. Interestingly, these authors also identify radioresistant glutamine-independent sublines, in which activation of autophagy in response to glutamine depletion serves as a pro-survival mechanism, likely providing metabolic substrates [37]. 

The cystine-glutamate antiporter xCT plays a major role in GSH synthesis pathway, by providing environmental cystine. The activity of xCT is essential for survival of a stem-like, CD44 variant (CD44v)-positive cell population in head and neck squamous cell carcinoma (HNSCC). In these stem-like cells, xCT inhibition by sulfasalazine treatment impairs GSH synthesis, leading to ROS-mediated cell death, without affecting CD44v-negative, differentiated cells in the same tumor [38]. Interestingly, Nagano and colleagues have more recently uncovered an interplay between ASCT2 and xCT affecting redox homeostasis in HNSCC stem-like cells. As described in the previous section, GLUD and xCT compete for glutamate, which can be either converted to αKG driving TCA cycle or exported in exchange for cystine. Nagano and colleagues identify a CD44v-positive CSC subset which express high levels of ASCT2 and display enhanced glutaminolysis and high TCA cycle activity. These ASCT2+/CD44vhigh stem-like cells are particularly sensitive to sulfasalazine treatment, because xCT inhibition dysregulates glutamate homeostasis by forcing excess glutamate towards the TCA cycle, thereby increasing mitochondrial metabolism and ROS levels [42].

Glutamine metabolism contributes to cellular redox homeostasis, not only by modulating GSH synthesis but also by maintaining a cytosolic pool of reduced NADPH. This NADPH pool acts as a donor of reductive potential for the regeneration of ROS-detoxifying enzymes and GSH [20]. Glutamine-derived NADPH has been shown to be required for survival of pancreatic cancer cell lines PANC-1 and SW1990 grown under CSC conditions (PCSC). In these cells, glutamine deprivation reduces expression of stemness-related genes, inhibits self-renewal, and impairs sphere formation. In line with a redox imbalance, PCSC spheres grown in glutamine-depleted conditions accumulate intracellular ROS. Interestingly, ROS accumulation can be prevented and rescued by supplementation with oxaloacetate [39]. It has been previously demonstrated that KRAS-mutated human pancreatic ductal adenocarcinoma (PDAC) reprogram glutamine metabolism and do not convert glutamate to αKG. Instead, PDAC cells transport glutamine-derived aspartate from mitochondria to the cytosol, where the aspartate transaminase GOT1 convert aspartate to oxaloacetate, finally used to generate malate and pyruvate. This series of reactions increases NADPH levels and maintains redox homeostasis [43]. Hence, this same pathway appears to be preferentially activated in PCSC, which indeed overexpress various transaminases. Accordingly, Li et al. demonstrate that transaminase inhibitors enhance ROS generation and promote radiation sensitivity in PCSC [39]. 

A central role for glutamine-derived NADPH has been demonstrated in non-small-cell lung cancer (NSCLC) H460 spheroids [44]. However, in this experimental system adaptation to anchorage-independent growth induces a fundamental change in citrate metabolism, with increased IDH1-dependent reductive carboxylation that promotes the formation of citrate from αKG in the cytosol. Production of citrate in the cytosol is linked to citrate flux to the mitochondria, where oxidative decarboxylation by IDH2 generates a mitochondrial NADPH pool and reduces mitochondrial ROS (Figure 2). The authors extend this observation to spheroids from multiple lung, colon and breast cancer cell lines. Although Jiang and colleagues do not investigate the impact of anchorage-independence on cancer cells stemness, previous studies have demonstrated that spheroids are enriched in stem-like cells, in comparison with adherent cultures [45,46,47]. Therefore, it is plausible to hypothesize a more general role for IDH1/2-mediated glutamine-derived NADPH in redox homeostasis in CSC spheroids. However, it is important to note that loss of matrix attachment is associated with a strong induction of ROS. Consequently, the activation of this IDH1-dependent pathway in spheroids may specifically develop to counteract detachment-dependent ROS accumulation. 

The activity of IDH1 enzyme in protecting CSC from oxidative damage has also been observed in glioma-initiating cells (GIC), where IDH1 inactivation, via silencing or pharmacological inhibition, diminishes NADPH production, reduces GSH levels, and increases ROS, thereby reducing GIC frequency [48]. Notably, oxidative decarboxylation of citrate by IDH1 produces NADPH and αKG, and the latter mediates indirect epigenetic effects of glutamine in CSC, as will be described below. On the whole, these studies show that IDH1-driven metabolic reprogramming is important for maintaining cancer stem cell compartment in different tissues. 

It is worth noting that in these studies CSC subsets have been identified through different approaches, such as sphere formation, expression of stem markers, and in vivo tumor initiation. The effects of redox imbalance in cancer stem-like cells ranges from promotion of a more differentiated cell state [46] to induction of caspase-dependent apoptosis [39], energy depletion-induced autophagy [35], and ferroptosis [36,42]. These differences are likely related to experimental kinetics, stem models used or, possibly, specific subsets of cancer stem-like cells analyzed by using different experimental approaches, and the tissue of origin. For instance, it has been demonstrated that TNBC have an increased susceptibility to ferroptosis [49], which may explain induction of ferroptosis in GD2+ breast cancer stem cells upon ASCT2 inhibition [36].

Interestingly, various studies have highlighted an indirect effect of glutamine depletion on cell signaling networks, mediated by redox imbalance. For instance, Liao et al. show that glutamine deprivation reduces the side population (SP) in A549 NSCLC cells through ROS-mediated inhibition of the β-catenin pathway [50]. SP is a subset of cancer stem-like cells characterized by elevated expression of transporters of the ABC family involved in active export of cytotoxic drugs out of the cell. Liao and colleagues show that the expression of ABCG2, as well as the expression of the stemness gene SOX2, is reduced in glutamine-starved SP. Sorting SP and non-SP cell subsets reveals an increased GSH content in stem-like SP cells and a time-dependent increase in intracellular ROS upon glutamine starvation. Similar effects are observed in two glioblastoma stem-like cell lines, GSC11 and GSC23, in which glutamine deprivation reduces neurosphere-forming ability. Mechanistically, increased intracellular ROS negatively regulates the β-catenin pathway by inducing β-catenin degradation and down-regulation of its canonical targets Survivin and Axin2. 

The connection between ROS overgeneration, inhibition of Wnt/β-catenin signaling and suppression of cancer stemness has also been demonstrated in hepatocellular carcinoma (HCC) [51]. In HCC stem-like cells, both GLS1 and Glutamate–Cysteine Ligase Catalytic Subunit (GCLC), the rate-limiting enzyme of GSH synthesis, are highly expressed, and associated with expression of multiple stem cell markers such as KFL4, SOX2, Nanog, Oct4, CD13 and CD44. In this model, GLS1 drives glutamine metabolism and GSH production, thereby reducing ROS levels and inducing nuclear translocation of β-catenin, which ultimately promotes stemness. Accordingly, glutamine deprivation or treatment with GLS1 inhibitors (C968 or BPTES) suppress the expression of CSC markers. Interestingly, this study highlights a positive regulatory loop controlling HCC stemness, in which Wnt/β-catenin promotes GLS1 expression, and GLS1-driven glutamine metabolism positively regulates Wnt/β-catenin signaling. 

On the whole, the studies described indicate an essential role for glutamine in protecting CSC from redox imbalance. In contrast with these studies, a signaling pathway linking glutamine deprivation to stemness induction has been proven in epithelial ovarian cancer cell lines PA1 and OAW42, and colorectal cancer cell line HCT116. Here, glutamine depletion increases mitochondrial ROS, which activate MAPK signaling cascade. ERK1/2 phosphorylate dynamin-related protein-1 (DRP1) at Ser616. Once phosphorylated, DRP1 can bind to the mitochondrial outer membrane, constrict the membrane and promote mitochondrial fragmentation. Fragmented mitochondria localized in the perinuclear region increase ROS locally and induce stem-like properties, as demonstrated by enhanced numbers of CD44 and CD117/CD45 positive CSC [52]. The causal link between ROS and DRP1 phosphorylation is supported by the ability of the ROS scavengers NAC and GSH to reduce both DRP1 phosphorylation and mitochondrial fragmentation in glutamine-deprived condition. Moreover, ERK inhibition prevents DRP1 phosphorylation in glutamine-deprived cells. Finally, these authors demonstrate that treatment with GLS1 inhibitor L-DON mimics the effects of glutamine starvation and enhances the expression of the stemness markers. Hence, these results suggest that regional glutamine deprivation may lead to the dedifferentiation of cancer cells. Interestingly, in this study redox imbalance does not result from reduced glutathione synthesis since no significant difference in cellular GSH is observed upon glutamine starvation. 

### 2.2. Glutamine, TCA Cycle, Anaplerotic and Cataplerotic Fluxes in CSC

An important function of glutamine metabolism in cancer is the replenishment of TCA cycle intermediates to support bioenergetics and biosynthesis. However, TCA cycle intermediates can also exit the cycle and contribute to glutamine-dependent cataplerotic pathways involved in cellular biosynthetic reactions. The balance between these two processes is mostly regulated by two enzymes: GLS, which converts glutamine to glutamate and initiates anaplerosis; and GS, which catalyzes de novo glutamine synthesis from glutamate and ammonia. Below, we will describe recent findings demonstrating how different glutamine-derived products serve different functions in different CSC populations. 

A key function of anaplerotic reactions is generation of ATP. This function appears to critically contribute to chemo-resistance in a stem-like cell line derived from HepG2 cells, characterized by a high level of stem markers CD49f, CD99, CD34 and overexpression of the ATP-binding cassette transporter P-glycoprotein, which confers it resistance to doxorubicin. In these cells, treatment with GLS inhibitor BPTES reduces P-glycoprotein activity and doxorubicin efflux, suggesting that resistant HepG2 stem-like cells depend on mitochondrial ATP production fueled by glutamine [53]. 

A different GLS-mediated biosynthetic pathway is NEAA synthesis; in fact, glutamine directly supplies nitrogen for asparagine synthesis, but also contributes to the synthesis of several amino acids through its conversion to glutamate [54]. A role for glutamine in driving NEAA biosynthesis has been described in CSC from gynecological malignancies. Here, metabolome analysis of OVTOKO (ovarian clear cell adenocarcinoma) and SiHa (cervical squamous cell carcinoma) cell lines grown under 3D spheroids conditions reveals an increased concentrations of amino acids related to TCA cycle, such as glutamate, aspartate, and serine. Interestingly, in this CSC model, ROS levels in 3D spheroids are lower than in cells in 2D adherent culture conditions, and glutamine does not appear to serve a major role in redox homeostasis [55]. 

Anaplerosis also plays an important role in glioblastoma (GBM). GBM stem-like cells (GSC) show a differential sensitivity to GLS inhibitors, which correlates with GLS expression itself. In GLS-high expressing GSC, treatment with the GLS inhibitors Compound 968 (C968) or CB839 reduces proliferation, induces a G1 cell cycle arrest, and severely impairs neurosphere-forming ability. Interestingly, in these stem-like cells GLS inhibitors affect neither GSH or GSSG concentrations, nor increases intracellular ROS. Instead, GLS inhibition reduces intracellular concentrations of the TCA cycle intermediate succinate, as well as glutamate-derived NEAA aspartate and alanine. According to a central anaplerotic role for glutamine, the anti-proliferative effect of CB839 on GLS-high expressing neurospheres is readily rescued by addition of either Glu or αKG [56]. Interestingly, these authors also demonstrate that the GLS inhibitor CB839 outperforms C968 in terms of enzymatic inhibitory activity. A role for glutamine as precursor for amino acids biosynthesis has been described in a different subset of GBM brain tumor stem cells (BTSC) [57]. This cell subset is characterized by low expression of the astrocytic glutamate transporters EAAT1 and EAAT2 (namely, excitatory amino acid transporters encoded by SLC1A3 and SLC1A2 genes, respectively). Inhibition of GLS in these cells depletes intracellular glutamate and triggers the amino acid deprivation response (AADR) pathway and apoptotic cell death. Hence, this BTCS subset demonstrates a unique metabolic dependence on glutamate to maintain intracellular amino acid levels, whereas BTCS expressing high levels of EAAT1 and EAAT2 are able to counteract glutamate depletion and are resistant to GLS inhibitors. Accordingly, pharmacologic block of EAAT transporters with L-trans-2,4-PDC [58] sensitizes GLS-resistant BTSC lines to CB839, and, conversely, treatment with the compound LDN-0212320 which increases EAAT2 protein levels [59] and rescues survival of GLS-sensitive BTSC upon CB-839 treatment. 

The results described in these latter works suggest an important role for GLS in diverse subsets of GBM stem-like cells. This suggestion is supported by the observation that oncogenic Notch signaling, which suppresses differentiation and sustains stemness in GBM [60], regulates both GLS1 expression and glutamate synthesis in GSC [61]. Accordingly, glutamine addiction is considered a key metabolic vulnerability in GBM [62]. 

In contrast to previous studies, Tardito and colleagues show that compared to differentiated cells, primary human GSC expressing stem markers CD133, Olig2 and Sox2, grow independently of glutamine supplementation [63]. Mechanistically, GSC uptake glutamate and upregulate GS which catalyzes conversion of glutamate to glutamine, used for purine biosynthesis. The central role of GS is substantiated by the ability of methionine sulfoximine (MSO), an inhibitor of GS, to abolish the growth of GSC in glutamine-depleted condition. Hence, in this GBM stem model, CSC rely on de novo glutamine synthesis and on glutamine-derived cataplerotic products. The discrepancies among these GBM studies might be related to differences in the culture media, since Tardito and colleagues employed a newly formulated medium containing nutrient concentrations comparable to human serum [63], whereas other authors utilized commercial media with a richer nutrient composition that may alter metabolic properties in cancer cells [64].

A relevant glutamine-derived cataplerotic product in CSC is represented by nucleotides. As just described, GSC overexpress GS and utilize glutamate-derived glutamine as nitrogen donors in purine biosynthesis [63]. The importance of glutamine-dependent nucleotide synthesis in GSC is supported by Wang and colleagues [65], who reveal specific upregulation of de novo purine synthesis in brain tumor initiating cells (BTIC). This metabolic alteration is necessary for BTIC maintenance since inhibition of purine biosynthesis abrogates BTIC growth, self-renewal and in vivo tumorigenesis. 

A critical role for GS in supporting stemness via nucleotides biosynthesis has also been demonstrated in liver cancer stem cells [66]. In this study, glutamine deprivation in Huh7 and HepG2 liver cancer cells induces upregulation of core stem markers OCT4, SOX2, and KLF4. Glutamine deprivation not only maintains stemness in pre-existing TIC but promotes the conversion of non-TIC OCT4-negative cells into OCT4-positive TIC cells. These TIC cells display high GS activity, which in turn increases cellular nucleotides content. Accordingly, pharmacological inhibition of GS abolishes stemness induction and sphere formation in glutamine-deprived conditions, which can be rescued by nucleotides supplementation. These authors confirm the above results in both primary cancer cells and patients’ tissue. For instance, patients-derived OCT4, CD133 or EpCAM-positive primary liver cancer cells display high GS expression and have stronger ability to form spheres in vitro and xenograft in vivo, as compared to primary cancer cells with low GS levels. Interestingly, analyses of fresh tumor samples from HCC patients demonstrate that glutamine concentration is lower in the core regions of the tumors than in the periphery, and that cells in core, glutamine-depleted areas overexpress OCT4. Finally, these authors demonstrate that upon glutamine deprivation mTORC2 is activated to promote HDAC3-mediated deacetylation and stabilization of GS.

A similar dependence on GS activity characterizes clonal human PDAC cells adapted to limiting concentrations of both glucose and glutamine [67]. These adapted cells exhibit an increased sphere-forming ability in vitro and tumor-forming capacity in vivo, thereby suggesting increased stemness. Notably, adapted clones share a common metabolic program, increasing both de novo glutamine synthesis through GS and nucleotide synthesis. In this cell model, mTORC1 activation prevents the proteasomal degradation of GS.

An important role for glutamine as nucleotide precursor has also been proposed in human and murine carcinoma CSC that evade from therapy-induced senescence (TIS) [68]. Cells that evade TIS represent a small CSC subset, characterized by plastic phenotype [69,70]. These CSC survive the cytotoxic impact of chemo- and radiotherapy in a dormant state but retain the potential to recover proliferation and ultimately contribute to tumor recurrence [71]. In line with high glutamine dependency, these cells overexpress ASCT2 and SNAT1 and are sensitive to glutamine transporters inhibitor L-γ-glutamyl-p-nitroanilide (GPNA). Importantly, in this CSC model, GS mediates resistance to glutamine ablation through nucleotides biosynthesis and allows evasion from TIS in glutamine-deprived conditions [68]. An essential role for glutamine metabolism in CSC that escape TIS is also suggested by the ability of the antineoplastic drug trabectedin to reduce both ASCT2 and GS protein levels, to decrease intracellular glutamine content, and to suppress TIS evasion [72]. Using a different approach based on the analysis of holoclones-forming cells [73,74] from MCF-7 breast adenocarcinoma these authors also demonstrate that glutamine deprivation specifically suppresses the growth of holoclones, while on the opposite, increasing glutamine concentration leads to an increase in the relative percentage of holoclones, further suggesting that undifferentiated, quiescent CSC at the apex of the hierarchy rely on glutamine to resume proliferation [68]. 

Finally, a different metabolic need boosts GLS-mediated glutamine catabolism in an interesting prostate cancer cell model in which stemness and epithelial–mesenchymal transition (EMT) properties are uncoupled [75]. In these CSC an enhanced susceptibility to acidic conditions assigns a specific function to glutamine-derived ammonia, released during GLS-mediated deamination. Analysis of main metabolic differences between epithelial-like CSC (e-CSC) and mesenchymal-like non-CSC subpopulations identifies an increased glucose consumption and a more robust Warburg effect in e-CSC. Accordingly, glucose deprivation or treatment with the glycolytic inhibitor 2-deoxyglucose hampers the sphere-forming ability of e-CSC. Interestingly, the contribution of glutamine to the synthesis of TCA cycle intermediates is higher in e-CSC, which also show higher expression levels of GLS1. Still, treatment with the GLS inhibitor BPTES inhibits e-CSC spheroids but cannot be rescued by supplementation with αKG. Instead, the key function of glutamine in e-CSC is to provide resistance to acidic conditions through GLS-dependent release of ammonia. Accordingly, incubation in acidic culture media demonstrate that e-CSC are more growth inhibited than mesenchymal-like non-CSC. Hence, this study reveals an important metabolic role for glutamine as ammonia precursor.

On the whole, these studies demonstrate that GLS-dependent glutamine catabolism supports diverse metabolic dependencies in CSC populations, which can be potentially targeted, but also highlight a dangerous role for GS enzyme that can be induced in cancer cells under nutrient stress, thereby driving cells toward a glutamine-independent stem-like cell phenotype.

### 2.3. Glutamine and Epigenetic Modifications in CSC

As previously mentioned, downstream of glutamine αKG mediates important effects on the epigenetic regulation of stemness genes and CSC differentiation. CSC are characterized by two distinctive properties, i.e., the ability to self-renew and to differentiate from early progenitors to fully differentiated progeny. CSC fate transitions depend on specific transcriptional programs, which are activated or silenced during the progression from precursors to differentiated progeny and from differentiated to less differentiated states. Epigenetic modifications control chromatin accessibility and critically contribute to state-specific gene expression. Major epigenetic mechanisms include DNA methylation, histone acetylation, histone methylation [76], and glutamine, through αKG, critically contributes to both DNA and histone methylation. 

Histone lysine methylation, which controls chromatin accessibility to transcription factors and so gene expression, is catalyzed by histone methyltransferases and reversed by histone demethylases. Generally, trimethylation of histone at lysine 9, 27 and 20 (H3K9me3, H3K27me3, H4K20me3) is associated with silenced chromatin states, whereas methylation of H3K4, H3K36, and H3K79 correlates with active transcription [77]. In addition, cytosine in DNA can be methylated to form 5-methylcytosine. Although high levels of cytosine methylation in promoter regions have been classically considered as a mark of silenced, non-transcribed genes, more recently, CpG methylation has been shown to affect transcription factor binding both positively and negatively [78]. The link between glutamine metabolism and histone methylation is αKG, which acts as a cofactor for a family of histone demethylases termed Jumonji C-domain-containing histone demethylases (JHDMs) [79]. In addition, αKG is also a cofactor for ten-eleven translocation (TET) methyl-cytosine dioxygenases, which oxidize 5-methylcytosines, thereby facilitating DNA demethylation and gene expression [80]. 

A critical role for αKG-dependent epigenetic modulation in CSC has been demonstrated in glioma-initiating cells, in which upregulation of wild-type IDH1 activates oxidative decarboxylation of citrate to increase αKG levels. In turn, αKG-dependent histone demethylases promote a more dedifferentiated, stem-like cell state [48]. Accordingly, inhibition of IDH1 by shRNA in glioma-initiating cells leads to increased histone trimethylation on H3K4, H3K9, H3K27, enhanced susceptibility to differentiation stimuli, and reduces stem cell frequency. 

The α-KG- and JHDMs-dependent histone H3 demethylation is also essential for maintenance of stemness gene expression in radioresistant, stem-like prostate cancer sublines. Accordingly, glutamine starvation of radioresistant prostate stem-like cells leads to increased trimethylation of histone H3 at lysine 27 (H3K27me3) and downregulation of genes involved in CSC maintenance [37]. 

A different picture is provided by Tran and colleagues, who used organoids derived from patients with colorectal cancer (CRC) and several in vivo CRC tumor models to show that αKG supplementation suppresses Wnt signaling and promotes cellular differentiation, thereby restricting cancer stemness [81]. Mechanistically, αKG supplementation promotes drastic DNA and histone hypomethylation at genes related to intestinal differentiation, thereby driving their transcriptional activation. Furthermore, αKG induces DNA demethylation and the transcriptional activation of various negative regulators of Wnt signaling pathway such as Dkk3, Dkk4 and Fat1. In line with these data, glutamine starvation in organoids induces an opposite effect, increasing expression and nuclear localization of β-catenin and expression of Lgr5 stem cell marker. Notably, by analyzing heterozygous APC-mutant (ApcMin/+) mice and ApcMin/+ small intestinal organoids, Tran and colleagues demonstrate that the effects of glutamine restriction on Wnt signaling and stemness is more profound in cells with predisposed genetic alterations. Hence, these results suggest that environmental glutamine restriction in tumor mass can hyperactivate Wnt signaling and induce a stem-like phenotye. 

A similar picture emerges in Ras-3T3 and M229 melanoma cells, where glutamine starvation induces histone hypermethylation and results in the upregulation of a panel of dedifferentiation genes (i.e., CD271, CD133 and ABCB5), thereby increasing stemness [1]. Notably, glutamine deprivation induces cancer cell fate conversion, and not expansion of a pre-existent subset of stem-like stem cells, as demonstrated by the dedifferentiation of FACS-sorted CD133-/CD271- double-negative cells into CD133+/CD271+ CSC in low glutamine media. Interestingly, these authors analyze the extent of glutamine heterogeneity regionally within xenograft tumors derived from KRasV12-3T3 cells, MDA-MB-231 TNBC, and V600EBRAF melanoma cells M229 and M249, and show that low glutamine concentrations in tumor core regions leads to decreased αKG levels, and a dramatic histone hypermethylation at H3K27me3. H3K27me3 has been shown to play a critical role in maintaining self-renewal and pluripotency in non-cancerous human embryonic stem cells [82]. Consistently, core regions of xenograft tumors display upregulation of stemness genes, while matched peripheral regions show increased differentiation markers. 

A heterogeneous glutamine distribution within the tumor mass has also been demonstrated in HT-29 colorectal cancer line xenografts, in which magnetic resonance imaging (MRI) shows that glutamine uptake is spatially localized within the tumor. In this study, however, expression of ASCT2, GLS, SNAT2, as well as expression of CSC markers CD44 and CD166, is increased in the area with high glutamine uptake, suggesting a positive role for glutamine in the induction of stemness [83].

On the whole, these studies demonstrate a critical role for glutamine-derived αKG in epigenetic reprogramming of CSC, but also reveal different effects on different CSC populations, activation of cell type-specific signaling pathways, and the need for future research. In addition, as stressed by Pan and colleagues [1], whether glutamine serves as a major source for αKG in vivo depends on both the tumor genotype and the tissue of origin.

### 2.4. Stem Marker Proteins and Glutamine Metabolism: Direct Interplay

In some cases, it is possible to envision a more direct link between the expression of specific stem cell markers and the activation of glutamine metabolism. For instance, a subset of PDAC tumor-initiating cells expresses high cell surface levels of stem marker CD9. These CD9+ stem-like cells display increased organoid formation capability and generate xenografts in vivo at limiting dilutions [84]. Interestingly, CD9 belongs to the tetraspanin family of membrane proteins which controls the spatial organization of biological membranes by forming large protein networks called tetraspanin webs [85]. In PDAC tumor-initiating cells, CD9 interacts with the glutamine transporter ASCT2 and promotes its plasma membrane localization, thereby directly enhancing glutamine uptake and replenishment of the TCA cycle. Interestingly, these authors also demonstrate that heterozygous deletion of CD9 (CD9WT/Δ) in PDAC organoids and tumors increases cell sensitivity to both GLS inhibitor CB-839 and to ASCT2 inhibitor V9302. In contrast, complete inactivation of tetraspanin CD9 (CD9Δ/Δ) activates a compensatory response, through SLC1A5 transcriptional upregulation [84]. 

Another example of a direct link between stemness-related surface markers and glutamine uptake machinery is represented by ganglioside GD2, which has been shown to be specifically expressed in small cell lung cancers (SCLC) [86]. Interestingly, analysis of GD2-associated molecules by EMARS-MS (enzyme-mediated activation of radical sources-mass spectrometry) in SCLC SK-LC-17 cells overexpressing GD2 reveals that GD2 associates with ASCT2 and recruits it to glycolipid-enriched microdomain/rafts, thereby directly enhancing glutamine uptake [87]. Thus, it seems plausible to hypothesize that a similar mechanism operates in the previously described GD2+ triple-negative breast cancer stem cell population, which depends on ASCT2-mediated glutamine uptake for GSH generation and redox homeostasis [36]. Interestingly, the CSC marker CD44 has also been identified among the candidate molecules associating with ganglioside GD2 in SK-LC-17 cells [87]. 

A causal relationship between glutaminase activity and ALDH levels has been proposed in head and neck squamous cell carcinoma (HNSCC) [88]. Specifically, Kamarajan and colleagues show that GLS1 drives ALDH expression. In this model, HNSCC stem-like cells (identified as CD44hi/ALDHhi) exhibit enhanced sphere-forming ability and elevated levels of GLS1 and intracellular glutamate. Inhibition of glutaminase activity, with either L-DON or shRNA-mediated gene silencing, suppresses tumorsphere formation in vitro and tumorigenesis in vivo, and, importantly, reduces ALDH1A1 expression both at mRNA and protein levels. Conversely, exogenous glutamine supplementation induces ALDH1A1 protein and drives conversion of CD44lo/ALDHlo into CD44hi/ALDHhi.

Finally, a role for CD133 cancer stem cell marker in modulation of thyroid cancer metabolism has been proposed, through CD133-dependent NF-kappaB-mediated induction of aspartate/glutamate transporter SLC1A3. In line, knock-down of SLC1A3 reduces glutamate content and inhibits self-renewal and tumorigenicity of CD133+ thyroid cancer cells [89]. Interestingly, it has been demonstrated that p53 promotes cancer cell proliferation and survival under glutamine starvation by inducing SLC1A3 expression. SLC1A3 sustains TCA cycle and promotes utilization of aspartate for de novo synthesis of glutamate, glutamine and nucleotides [90].

Altogether, the studies described in these sections reveal a complex role for glutamine in regulating stemness and differentiation, where distinct glutamine downstream products, pathways and enzymes are activated in different CSC subpopulation. Main glutamine metabolic pathways described, as well as their effects on cancer stemness, are summarized in Table 1.

## 3. Glutamine Metabolism in Tumor Microenvironment: Tumor-Stroma Crosstalk Might Regulate CSC

**Figure 3:**
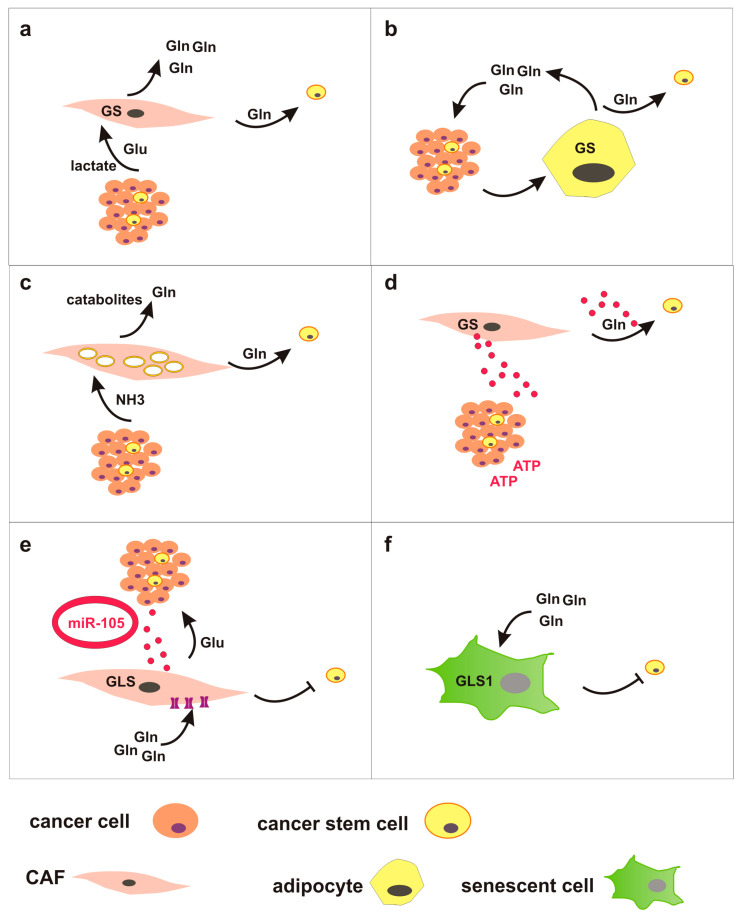
TME components may locally affect glutamine concentration and availability, thereby modulating neighboring CSC. (**a**) Crosstalk between CAF and cancer cells in the TME. CAF upregulate GS and under glutamine-deprived conditions sustain proliferation of glutamine-dependent cancer cells. On the other side, cancer cells release lactate and glutamate to support CAF metabolism. Glutamine produced by CAF might be available for CSC. (**b**) Crosstalk between adipocytes and cancer cells in the TME. High glutamine demand in cancer cells results in glutamine depletion within the TME and induces GS upregulation in dysfunctional cancer-associated adipocytes. Glutamine produced by adipocytes might be available for CSC. (**c**) Crosstalk between CAF and cancer cells in the TME. Ammonia produced by cancer cells selectively induces autophagy in CAF, leading to release of catabolites, including glutamine, which are taken up by cancer cells. Glutamine produced by CAF might be available for CSC. (**d**) Exosomes-mediated crosstalk between CAF and cancer cells in the TME. CAF-derived exosomes contain metabolites, including glutamine, which can be used by cancer cells. Glutamine-containing exosomes produced by CAF might be available for CSC. (**e**) Exosomes-mediated crosstalk between CAF and cancer cells in the TME. Exosome-encapsulated miR-105, released by cancer cells, induces upregulation of GLS and SLC1A5 in CAF. High glutamine demand in CAF can reduce glutamine availability for CSC. (**f**) Senescent cells upregulate GLS1 and rely on glutaminolysis for survival. Senescent cells can avidly uptake glutamine and reduce glutamine availability for CSC.

CSC in vivo are surrounded by differentiated cancer cells, as well as by several cellular and non-cellular components of the tumor microenvironment (TME). Non-cellular elements of the TME comprise various extracellular matrix proteins, whereas cellular components include numerous noncancerous cells such as fibroblasts, endothelial cells, adipocytes and immune cells. These stromal cellular components frequently show metabolic alterations, induced by the neighboring cancer cells. The metabolic crosstalk between tumor and TME components is emerging as a key regulator of cancer growth, metastasis and response to therapy. In particular, reprogramming of glutamine metabolism has been described in some stromal cells, which thereby contribute to control glutamine concentrations in the TME. Here, we briefly describe glutamine-related metabolic changes in cancer-associated fibroblasts (CAF), adipocytes, and senescent cells, and propose that these changes may indirectly affect CSC fate by modulating glutamine availability (Figure 3).

It is worth recalling that important metabolic interplay in the TME occurs between tumor cells and immune cells, where cancer-dependent glutamine depletion severely affects immune cells [91]. However, this topic is not discussed here since our knowledge about the ability of immune cells to modulate glutamine availability in the TME is still limited. 

CAF are the most abundant cells in the TME and play a prominent role in glutamine-related metabolic crosstalk with cancer cells. For instance, in high-grade serous ovarian adenocarcinomas CAF display high expression of glutamine metabolism-related genes, especially GLUL which catalyzes de novo glutamine synthesis, and various transaminases that can promote glutamate and glutamine biosynthesis [92]. Under glutamine-deprived conditions, these CAF sustain proliferation of glutamine-dependent cancer cells. Importantly, high glutamine metabolism in CAF is supported by lactate and glutamate released by cancer cells, thus revealing a metabolic symbiosis in the TME. 

A similar metabolic crosstalk has been demonstrated in peritoneal carcinomatosis of colorectal cancer between tumor cells and adipocytes, which represent a major component of the tumor microenvironment in peritoneum. In this TME model, deregulated uptake of glutamine by tumor cells induces GS upregulation in dysfunctional cancer-associated adipocytes and adipocyte-derived glutamine promotes resistance to chemotherapy in CRC cells via mTOR activation [93]. 

A role for microenvironmental ammonia in cancer drug resistance has been described by Ko and colleagues [94]. Ammonia, produced during the deamination of glutamine, is known to induce autophagy [95]. Here, ammonia produced by cancer cells selectively induces autophagy in CAF, leading to release of catabolites including glutamine, which are taken up by cancer cells. In turn, glutamine metabolism in cancer cells sustains bioenergetics and biosynthesis, but also generates ammonia which drives autophagy in stromal cells. This metabolic loop protects breast cancer cells from apoptosis under both baseline conditions and during treatment with tamoxifen [94].

CAF can also modify the microenvironment through exosomes. CAF-derived exosomes contain metabolites including glutamine, which can be used by cancer cells in nutrient-deprived conditions. For instance, exosomes released from patient-derived prostate CAF are internalized by prostate cancer cells, and they increase glutamine-dependent IDH-mediated αKG reductive carboxylation to generate citrate and supply TCA cycle in reverse manner [96]. Likewise, cancer cells can reprogram CAF metabolism via exosomes. Exosome-encapsulated miR-105, released by breast cancer cells, activates c-Myc signaling in CAF. Dysregulated c-Myc in CAF upregulates GLS and SLC1A5, enhances both glycolysis and glutaminolysis, and increases the secretion of metabolic intermediates such as glutamate to feed neighboring cancer cells. Interestingly, in nutrient-deprived conditions these CAF detoxify high concentrations of ammonia in the microenvironment by converting ammonium into amino acids. Hence, CAF can support cancer growth producing metabolic intermediated, but also by converting wastes/byproducts into metabolites used by cancer cells [97].

Glutamine-dependent metabolic interaction in the TME may also occur between different cancer cell subtypes. For instance, in breast tumor luminal-type cells overexpress GS and acquire independency from exogenous glutamine supply. In contrast, basal cells, due to dysregulated c-Myc signaling, overexpress GLS and are glutamine addicted. These subtypes grown in co-culture establish a metabolic symbiosis by which glutamine produced by luminal cells can support basal cells metabolism in glutamine-deprived conditions [25]. Interestingly, glutamine limitation induces GS upregulation in senescence-associated breast cancer stem cells that evade from TIS independently from the molecular subtype [68].

Other cellular elements of the TME heavily dependent on glutamine are senescent cells. Several stimuli can concur to induce senescence in the TME, such as oncogene activation, oxidative stress, hypoxia, chronic inflammation [98], and cancer therapies [71]. Furthermore, senescence can be induced both in tumor cells and in stromal cells. Interestingly, it has recently been shown that senescent cells upregulate GLS1 and rely on glutaminolysis for survival. Mechanistically, senescent cells display intracellular acidosis owing to lysosomal membrane damage and leakage of lysosomal H+ into the cytosol. GLS-dependent production of ammonia neutralizes acidic conditions and enhances survival of senescent cells [99]. Similarly, analysis of the metabolic requirements of TIS cancer cells demonstrates a preferential use of glutamine over glucose as mitochondrial fuel under nutrient-limiting conditions [100]. 

Finally, it has recently been shown that non-cellular components of the TME can modify cancer cells’ metabolism. For instance, extracellular hyaluronic acid increases glutamine uptake in breast cancer cell lines and in CSC in vitro [101]. 

On the whole, these studies show that diverse TME elements can uptake and restrain, or produce and release, glutamine in the microenvironment. We envision that these TME components may locally affect glutamine concentration and availability, thereby modulating neighboring CSC (Figure 3). For instance, it is plausible to hypothesize a competition between glutamine-dependent chemotherapy-induced senescent cells in the microenvironment and senescence-associated CSC that rely on glutamine for escaping from TIS. Reciprocal metabolic communication between TME components and CSC deserves further investigation.

## 4. Conclusions

In this review we have provided a summary of glutamine’s effect on CSC in solid tumors, highlighting the complex metabolic heterogeneity of this cellular compartment. CSC are composed of functionally different subsets, which differ in cell cycle status, differentiation potential, expression of stem-specific markers and, expected, metabolic phenotypes. Targeting glutamine metabolism has been proposed as a new therapeutic opportunity in cancer, and inhibition of glutamine metabolism can indeed inhibit self-renewal and decrease stemness in CSC. Nevertheless, plasticity can protect CSC and lead to resistance, for instance through the upregulation of glutamine producing enzymes and pathways, and development of glutamine-independent CSC subsets. Plasticity also allows the dynamic switch between CSC and non-CSC states, which correspond to different metabolic phenotypes. Recent development of single-cell gene expression profiling technologies and metabolomics offers the opportunity for dissecting the CSC compartments and may help to define targetable metabolic traits and vulnerabilities. 

We have also provided examples of glutamine-mediated crosstalk and symbiotic relationship between cancer cells and stromal components in the tumor microenvironment, suggesting that metabolic reprogramming in the TME likely influences CSC by locally limiting or increasing glutamine availability. More physiological culture media should be employed in vitro, and reliable in vivo models need to be developed to investigate metabolic CSC interactions in the TME.

## Figures and Tables

**Table 1:** Main glutamine metabolic pathways activated in CSC, their effects on cancer stemness, stemness markers.

Cell Type	Gln-Dependent Metabolic Pathway	Impact on Stemness	Stemness Markers	References
Embryonal carcinoma (EC)	GSH biosynthesis	+	Oct4, Nanog, Sox2 Sphere formation	[35]
Triple-negative breast cancer (TNBC)	GSH biosynthesis	+	GD2 Sphere formation	[36]
Prostate cancer	GSH biosynthesis	+	ALDH activity Sphere formation Tumor initiation	[37]
Head and neck squamous cell carcinoma (HNSCC)	GSH biosynthesis	+	CD44v Sphere formation Tumor initiation	[38]
Pancreatic ductal adenocarcinoma (PDAC)	GOT1-dependent NADPH biosynthesis	+	CD44, CD133, ESA, ALDH1 Sphere formation Tumor initiation	[39]
Head and neck squamous cell carcinoma (HNSCC)	glutaminolysis and TCA cycle	+	CD44v	[42]
Non-small-cell lung cancer (NSCLC)	IDH2-dependent NADPH biosynthesis	+	Sphere formation	[44]
Glioblastoma (GBM)	IDH1-dependent NADPH biosynthesis	+	Sphere formation Tumor initiation	[48]
NSCLC, GBM	GSH-mediated β-catenin stability	+	Side population Sox2, ABCG2 Tumor initiation	[50]
Hepatocellular carcinoma (HCC)	GSH-mediated β-catenin nuclear translocation	+	Oct4, Nanog, Sox2, CD44, CD133, KLF4 Sphere formation	[51]
Epithelial ovarian cancer colorectal cancer (CRC)	ROS-mediated ERK1/2-dependent DRP1 activation	−	Oct4, Sox2, Nanog, ABCG2, CD44 ALDH activity Sphere formation	[52]
HCC	mitochondrial ATP production	+	P-glycoprotein CD49, CD99, CD34	[53]
Ovarian clear cell adenocarcinoma (OCCA) Cervical squamous cell carcinoma (CSCC)	Amino acids biosynthesis	+	Sphere formation	[55]
GBM	TCA cycle and amino acids biosynthesis	+	Sphere formation	[56]
GBM	Amino acids biosynthesis	+	Sphere formation	[57]
GBM	Glutamate biosynthesis	+	Sphere formation	[61]
GBM	Nucleotides biosynthesis	+	CD133, Sox2, Olig2	[63]
GBM	Nucleotides biosynthesis	+	Sox2, NES, Olig2 Sphere formation Tumor initiation	[65]
HCC	Nucleotides biosynthesis	−	Oct4, Sox2, KLF4, CD133 Sphere formation	[66]
PDAC	Nucleotides biosynthesis	−	Sphere formation Tumor initiation	[67]
Breast carcinoma NSCLC	Nucleotides biosynthesis	+	CD44, CD24 Clonogenicity assay	[68]
PDAC	NH4+ production	+	Sphere formation	[75]
GBM	IDH1-dependent αKG biosynthesis	+	Sphere formation Tumor initiation	[48]
GBM	αKG-dependent histone demethylation	+	Sphere formation Tumor initiation	[48]
Prostate cancer	αKG-dependent histone demethylation	+	ALDH activity Sphere formation Tumor initiation	[37]
CRC	αKG biosynthesis	−	Lgr5 Organoid formation Tumor initiation	[81]
Melanoma TNBC	αKG biosynthesis	−	CD271, ABCB5, CD133, Nanog, Sox2, NES, KLF4	[1]
PDAC	TCA cycle	+	CD44 Organoid formation Tumor initiation	[84]
HNSCC	Glutamate biosynthesis and	+	CD44, ALDH	[88]

+ indicates induction of stemness, – indicates dedifferentiation or loss of stem cells.
